# Thinking in three dimensions: a different point of view for understanding autism

**DOI:** 10.3389/fnhum.2015.00484

**Published:** 2015-09-08

**Authors:** Gabriel Tzur

**Affiliations:** ^1^Behavioral Science, Ruppin Academic CenterEmek Hefer, Israel; ^2^Association for Children at RiskTel Aviv, Israel

**Keywords:** autism, ASD, prefrontal cortex, sensorimotor, integration, TOM, regulation

Autism spectrum disorder (ASD) is a complex phenomenon. This neurodevelopmental condition is characterized by a hallmark of impairments in social interaction, communication, and restricted activity. Neurocognitive studies in the last three decades provide important insights regarding the pathological development, and the complexity of the ASD condition (Frith, 1989; Belmonte et al., 2004; Baron-Cohen, 2006; Happe and Frith, 2006; Markram and Markram, 2010; Philip et al., 2012; Schore, 2013; Tang et al., 2014; Hahamy et al., 2015).

In this opinion, I discuss the *frontal integration model of ASD* (Ben Shalom, 2009), as a simplified model for understanding autism. I begin by a brief sketch of Ben Shalom's model. Then, I make links between Ben Shalom's model and central neurocognitive theories. I suggest that these novel links mutually elaborate both Ben Shalom's original account as well as these theories. This elaboration offers a three-dimension model that is consistent with frameworks that address neuropsychoanalytic developments in autism, such as developmental deficits in forging a unitary sense-of-self (Schore, 2013).

According to the *frontal integration model of ASD* (Ben Shalom, 2009), neurocognitive processing can be divided into three-levels: (1) a *basic-level* involving primary cognitive, emotional, and sensorimotor processing. For example, a loud unexpected sound that is perceived in primary auditory-sensory systems might trigger physiological emotional-responses, such as, fear (fast heart beats, etc.). (2) an *integrative-level*, that combines the output of all primary processes from the basic-level, and forms a global-coherent meaning, experience, or behavior. For example, the mental representation of the various primary elements that constitute the fear emotional response results in a conscious feeling of being afraid. (3) a *logical-level*, which forms abstract logical rules (if-then rules) from the basic-level. For example, “if I have fast heart beats and cold sweat, then I might be afraid.” This three level architecture is applied to four general psychological domains: emotion, memory, sensation-perception, motor.

Ben Shalom (2009) argues that some core ASD abnormalities are the result of deficits in medial-prefrontal cortex. These deficits are manifested in impairments in level-2 *integrative*-processes, which are essential for abilities, such as theory of mind (TOM), emotional-regulation and motor-planning. Moreover, this model suggests that intact level-3 *logical*-processes, can access level-1 basic-processes, and compensate for the deficits in level-2 *integrative*-processes. This type of compensation may assist ASD individuals in understanding and coping with the world around them. For example, an ASD individual with deficits in level-2 integrative-processes may fail feeling typical empathy toward a sad person. However, the ASD individual may rely on intact level-3 processes in order to recognize the emotional state, by forming a logical if-then rule, such as “if a person is crying, then he might be sad.” Nevertheless, this compensatory logical mechanism cannot fully cover for the core deficits in level-2 integrative processes. For example, in the absence of integrative-intuitive abilities, such as TOM, compensatory rule-based thinking and behavior often appears rigid and unflexible, accompanied with problems in understanding nuances of complex social situations. Consider an ASD individual that tries to apply the rule: “if someone said something that made others laugh then it is a joke, and I should laugh too.” While in this situation this ASD individual might laugh, he may not understand why the joke was funny, or may be somewhat confused if some people appear to be crying because they wipe tears from their eyes.

Several existing theories of ASD can be linked to key elements in Ben Shalom's (2009) model. First, the idea that ASD abnormalities relate to deficits in *integrative*-processes, aligns with the *central-coherence theory* (Frith, 1989; Happe and Booth, 2008). According to this theory, ASD individuals display weak central-coherence, that is, a reduction in integrative-processes that pull together large amounts of information into coherent wholes. This theory rests on early concepts of the Gestalt movement, which claimed that perceptual organization is the result of a top-down configuration of the whole, rather than a bottom-up sum of its parts. That is, “*the whole is greater than the sum of its parts*” (Aristotle). The weak central-coherence theory is supported by numerous studies showing that ASD individuals present better *local*-processing than *global*-processing. That is, greater ability to segment the whole design into its component parts, but impaired ability to perceive an integrated-coherent whole (i.e., not seeing the forest for the trees).

Ben Shalom's (2009) idea of impaired integrative-processes is not only consistent with the central-coherence theory, but it can also enrich it, by suggesting that weak central coherence is not limited to perceptual organization, but rather it can also be seen in three other psychological domains: *emotion* (e.g., deficits in forming integrative-conscious feelings to self or other), *memory* (e.g., deficits in the integrative-processes that constitute episodic memory, such as the ability to mentally travel back in time during recollection), and *motor* (e.g., deficits in integrating basic information from several modalities required for motor planning).

Second, Ben Shalom's premise of deficits in level-2 *integrative*-processes together with compensatory level-3 *logical*-processes is also congruent with the *empathizing-systemizing (E-S) theory* (Baron-Cohen, 2006). According to this account, ASD individuals show deficits in *empathizing* (i.e., hypo-empathizing), and an intact or superior ability in *systemizing* (i.e., hyper-systemizing). The term *empathizing* encompasses a range of terms, including TOM, and empathy (Baron-Cohen, 2004, 2006). Specifically, ASD deficits in empathizing, often linked to the medial-prefrontal cortex (Baron-Cohen and Belmonte, 2005), are consistent with Ben Shalom's ideas of medial-prefrontal cortex deficits in level-2 *integrative*-processes. Moreover, the E-S theory suggests that deficits in empathizing may range in severity (Baron-Cohen, 2006), and as such it extends Ben Shalom's model that concentrates on a general dysfunctionality.

Furthermore, the ASD *systemizing* idea suggested by the E-S theory (Baron-Cohen, 2006), aligns with Ben Shalom's idea of *logical* rule-based thinking (level-3). According to this idea, *systemizing* involves the formation of rules that are based on examining whether repeated application of a particular operation to a certain input, leads to a similar output. This theory posits that the human brain's ability to systemize can vary along eight different levels, from *hypo-systemizing* (low or non-ability to formulate rules), to *hyper-systemizing* (high ability to analyze and formulate rules).

Hyper-systemizing at the highest level may represent an outstanding unique ability, such as phenomenal ability to calculate numbers, or to analyze and compose music—i.e., *savant*-abilities. It has been also suggested, that *savant*-abilities operate by directly accessing low-level, less-processed information that exists in all human brains, but is not normally available to conscious awareness (Snyder, 2009). This idea supports Ben Shalom's suggestion regarding level-3 logical-processes that can compensate for deficits in level-2 integrative-processes, by accessing level-1 basic-processes.

Ben Shalom's idea that rule-based processing may interact with basic-processing, is consistent with the E-S theory that relates *hyper-systemizing* to an increased basic sensory sensitivity (Baron-Cohen, 2006). In this sense, the E-S theory expands Ben Shalom's model by suggesting that ASD abnormalities are not exclusively related to level-2, and can also be seen in level-1 *basic*-processes. These level-1 deficits may present different types of abnormalities, such as sensory *hyper-sensitivity* (e.g., covering ears against loud, unexpected sounds) and *hypo-sensitivity* (e.g., failure to react to pain) (Kern et al., 2007; Miller et al., 2007; Ben-Sasson et al., 2009; Lane et al., 2010; Lloyd et al., 2013).

Third, level-1 deficits in ASD are consistent with the *intense world theory* (Markram and Markram, 2010). According to this theory, hyper-functioning, that is, high-local connectivity within neural assemblies (Belmonte et al., 2004), may lead to excessive flow of information from *sensory* areas to *higher integration* areas, causing overload that can lead to intense experiences, and fragmented understanding of the world (Markram and Markram, 2010).

The integration between Ben Shalom's (2009) model and the aforementioned central neurocognitive theories, suggests that ASD abnormalities can be related to each level of Ben Shalom's model (1-basic, 2-integrative, 3-logical), and each level can be seen as a broad *dimension* that holds multiple types of processes that can vary in their functionalities. Furthermore, different potential deficits *within* each dimension, deficits *between* interacting dimensions as well as compensation between dimensions, demonstrate the complexity of the autism spectrum condition. For example, within the *basic-dimension* a differential profile of sensorimotor deficits can include hyper/hypo-sensitivity in auditory, visual, tactile, smell, somatic, and vestibular processes. These differential basic-dimension deficits may interact with different types of global-integration process deficits in the *integrative-dimension*, and with different types of compensatory high-cognitive processes in the *logical-dimension* (e.g., high ability to formulate rules).

This *hierarchical* three-dimensional model also aligns with neuropsychoanalysis frameworks that address the developmental pathology of social-emotional skills in autism. According to the *regulation theory*, the bi-directional mother-infant emotional-communication serves as a mutual psychobiological emotion-regulation process that is the basis of all later social-emotional skills (Schore, 2013). These mother-infant mutual interpersonal emotion-regulation processes begin with early unconscious emotional communications that are based on primary *sensorimotor* processes (e.g., visual-facial, auditory-prosodic, tactile-gestural). Later in development, these *basic* interpersonal regulatory processes become more holistically *integrated*, enabling the emergence of intrapersonal self-regulation and a coherent sense-of-self (Schore, 2013; Schore and Schore, 2014).

The regulation theory suggests that the infant's social-emotional development is manifested in a *hierarchical* development of interconnected *right-brain-limbic* areas. For example, in the basic-dimension, the right *amygdala*, which is functional at birth, is responsible for various rudimentary elements of emotional-communication, such as production/recognition and arousal-regulation. Importantly, the right amygdala, which is characterized as “a hub of a network” (Markowitsch and Staniloiu, 2011), forms novel connections with multiple integrative self-regulatory regions including the right *anterior-cingulate*, and the right *orbitofrontal cortex*. These regions are responsible for *integrative*-intuitive assessment of complex social situations (Schore, 2013).

The regulation theory posits that basic mother-infant emotional-communication can either facilitate or inhibit the maturation of the infant's self-regulatory processes and social-emotional skills (Schore, 2013). In this sense, deficits in the infant's *basic-dimension*, such as hyper-aroused amygdala (Schumann et al., 2009), and hyper/hypo-sensitivity in primary sensorimotor processes (Ben-Sasson et al., 2009; Lane et al., 2010; Lloyd et al., 2013), may relate to an intense dysregulated emotional state (Markram and Markram, 2010), in which mother-infant sensorimotor emotional-communication is disrupted. These disruptions result in deficits in the infant's integrative dimension development. Deficits between these interacting dimensions, may explain the autistic infant's dissociative withdrawal from the intense emotional world around him (Markram and Markram, 2010; Schore, 2013), and the autistic's need for sameness of environment and activities.

According to the three-dimensional model, repetitive activities may relate to different deficits and compensatory processes within and between dimensions. This illustrates the idea that Ben Shalom's model is non-deterministic, allowing different etiologies to similar symptoms. For example, echolalia (i.e., repetition of phrases, words or parts of words), may serve a self-regulatory function (Prizant and Duchan, 1981), by helping ASD children gain control over their experience when there is intense sensory-emotional arousal in the *basic-dimension*. At the same time, echolalia may also relate to compensatory processes in the *logical-dimension*, because repetitions are needed to formulate logical-rules, or serve as a way of communication (Prizant and Duchan, 1981), as if saying “I heard what you said, and I'm still processing it.”

Thinking in these three-dimensions offers a simplified model that may help in understanding autism across different theoretical frameworks and therapeutic techniques. For example, addressing the *basic-dimension* and assessing primary sensorimotor processes are most important when assisting the infant or the child to regulate (or work around) his emotional-sensorimotor experience. These intense emotional-sensorimotor experiences may relate to bodily terrors in the autistic psychic experience, such as “liquefying/burning/freezing,” and “losing part of the body,” when experiencing physical separation from the caregivers (Tustin, 1972, 1986). Early intervention, that facilitate mother-infant social-emotional communication, and mental connections between emotions, feelings, thoughts, sensations and behavior, may facilitate neural connections (Sullivan et al., 2014). Moreover, assessing the *logical-dimension*, and nurturing rule-based abilities that can compensate for impairments in the integrative-dimension, may help ASD individual to cope better in their surroundings and even excel in their fields of interest.

This opinion invites researchers and clinicians to further elaborate this three-dimensional model. Addressing distinct profiles of deficits *within* and *between* dimensions may help to assess and assist the developmental course of ASD individuals.

## Conflict of interest statement

The author declares that the research was conducted in the absence of any commercial or financial relationships that could be construed as a potential conflict of interest.
